# Short and long time effects of low temperature Plasma Activated Media on 3D multicellular tumor spheroids

**DOI:** 10.1038/srep21421

**Published:** 2016-02-22

**Authors:** Florian Judée, Céline Fongia, Bernard Ducommun, Mohammed Yousfi, Valérie Lobjois, Nofel Merbahi

**Affiliations:** 1 Université de Toulouse ; UPS, INP ; LAPLACE; 118 route de Narbonne, F-31062 Toulouse, France; 2 CNRS ; LAPLACE; F-31062 Toulouse, France; 3 Université de Toulouse; ITAV-USR3505, F-31106 Toulouse, France; 4 CNRS; ITAV-USR3505, F-31106 Toulouse, France; 5 CHU de Toulouse; F-31059 Toulouse, France

## Abstract

This work investigates the regionalized antiproliferative effects of plasma-activated medium (PAM) on colon adenocarcinoma multicellular tumor spheroid (MCTS), a model that mimics 3D organization and regionalization of a microtumor region. PAM was generated by dielectric barrier plasma jet setup crossed by helium carrier gas. MCTS were transferred in PAM at various times after plasma exposure up to 48 hours and effect on MCTS growth and DNA damage were evaluated. We report the impact of plasma exposure duration and delay before transfer on MCTS growth and DNA damage. Local accumulation of DNA damage revealed by histone H2AX phosphorylation is observed on outermost layers and is dependent on plasma exposure. DNA damage is completely reverted by catalase addition indicating that H_2_O_2_ plays major role in observed genotoxic effect while growth inhibitory effect is maintained suggesting that it is due to others reactive species. SOD and D-mannitol scavengers also reduced DNA damage by 30% indicating that 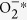 and OH^*^ are involved in H_2_O_2_ formation. Finally, PAM is able to retain its cytotoxic and genotoxic activity upon storage at +4 °C or −80 °C. These results suggest that plasma activated media may be a promising new antitumor strategy for colorectal cancer tumors.

Low temperature plasmas are weakly ionized gases involving various active species (reactive species, charged particles, long lived time excited species, UV radiation and also localized electric fields) diluted in neutral background gas at ambient temperature. Several different setups of low temperature plasma jets at atmospheric pressure can be found in the literature as for instance dielectric barrier discharges (DBD) monitored by pulsed or AC voltage^1^,^2^,^3^, corona discharges powered by DC or AC supply^4^,^5^, Radio-Frequency reactor^6^ and Microwave plasmas^7^,^8^,^9^. Several carrier gases can be used to produce low temperature plasmas such as helium, argon, nitrogen, air or mixture of inert gases with air or N_2_ or O_2_, or H_2_O, etc. Complex chemical kinetics is triggered by low temperature plasmas operating in open air at quasi-ambient temperature. The dominant radical species are likely to be formed from oxygen and nitrogen molecules leading to reactive oxygen species (ROS) and reactive nitrogen species (RNS), although other oxides due to the presence of humidity such as hydroxyl, hydrogen peroxide and hydroperoxyl radicals can also be produced.

Low temperature plasmas application to medicine has received increasing interest over the last decade^10^,^11^,^12^,^13^,^14^,^15^,^16^,^17^. In the case of neoplasia, low temperature plasmas operating in open air have been successfully tested, both *in vitro* and *in vivo*^18^,^19^,^20^,^21^,^22^,^23^,^24^,^25^,^26^. Recently, studies based on low temperature plasma conditioned culture media usually called Plasma Activated Medium (PAM) have demonstrated their activity on cancer cells. *In vitro*, PAM was shown to stop proliferation and induce DNA damage in HCT116 colon cancer cells^20^, to induce apoptosis on glioblastoma brain tumor cells^27^, to inhibit proliferation on human lung adenocarcinoma epithelial A549, human hepatocarcinoma HepG2 and mammary adenocarcinoma MCF-7 cells^28^ and chronic chemo-resistant ovarian cancer cells^29^. *In vivo*, subcutaneous injection of PAM in murine tumor was reported to induce cytotoxicity against ovarian clear cell carcinoma^30^ and suppress choroidal neovascularization in mice^31^. However, the active mechanisms and the identity of the chemical byproducts generated during conditioning of the culture media by the plasma and involved in these reported effects are either not well understood and nor clearly identified and quantified.

In the present study, we investigated the effects of liquid culture media conditioned during a short exposure time by pulsed He plasma jet on growth inhibition and the genotoxicity on HCT116 colorectal multicellular tumor spheroids (MCTS). The MCTS is a 3D culture model known as an intermediate stage in elaboration of anti-cancer therapy between classical *in vitro* (monolayer culture cells) and *in vivo* models^20^,^32^. We investigated more particularly the penetration effects of PAM not only on the outermost cells of MCTS but also inwards the inner regions. We also identified and quantified the specific role of H_2_O_2_ and also the species involved by using several scavengers (catalase, SOD, D-mannitol and L-histidine) on DNA damage. Finally, we explored PAM stability and genotoxic and cytotoxic effects on MCTS upon storage at different temperatures and for duration varying from hours to several days.

## Results

### PAM retains genotoxic and cytotoxic effects in time transfer dependent manner

**Figure 1:**
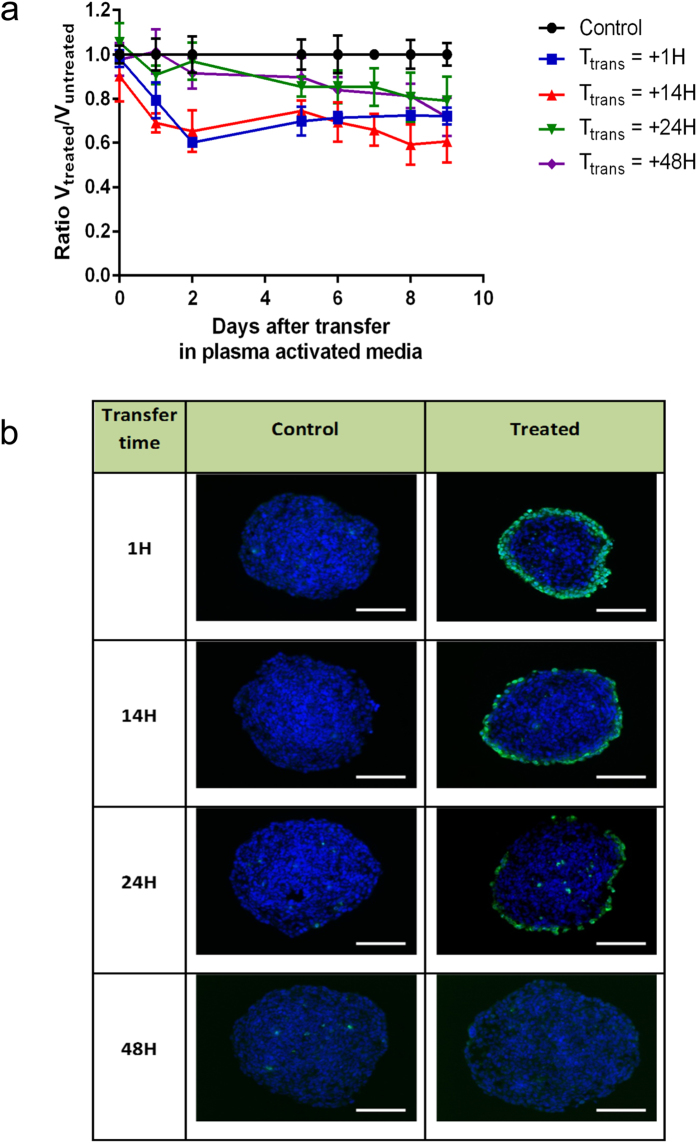
Growth inhibition and genotoxic effects of PAM on HCT116 MCTS. (**a**) Relative growth of spheroid after PAM treatment (Texp = 120 s) for several transfer times Ttrans. The spheroids were cultured during five days in DMEM and transferred in PAM at day 0. Data are shown as means ± SD from four independent experiments. (**b**) Genotoxic effect of PAM detected 4 h after transfer time of MCTS in PAM. Immuno-detection of the phosphorylation of the histone variant H2AX (phospho-H2AX, green) on 5 μm cryosections of control spheroids or treated spheroids. DAPI (blue) correspond to the Nuclei. Data shown are representative images from four independent experiments. Scale bar, 100 μm.

We first considered a duration of exposure (Texp) of 120 s to generate PAM. HCT116 colon carcinoma multicellular tumor spheroids were transferred in PAM after increasing delay subsequent to the activation of culture media by the plasma jet (transfer times of 1 h, 14 h, 24 h and 48 h) and their growth was evaluated. Figure 1a displays the daily volume ratio between treated and untreated spheroids monitored during 9 days. Control spheroids, not exposed to PAM, grew as expected during the time of analysis (normalized ratio is 1). In contrast, growth of the spheroids cultivated in PAM was inhibited by about 40% in the case of short transfer times (1 h or 14 h). Growth inhibition appears to be dependent on the time transfer of the spheroid in PAM after preparation and indicates that two phases are involved in this inhibition. The first one occurs during the first day with a rapid decrease of MCTS volume followed by slower volume decrease the next days. For longer transfer times, spheroid volume decreases continuously and reaches about 20% of loss 9 days after spheroid transfer in PAM.

In order to assess DNA damage induced by PAM, spheroids were exposed after the same transfer times (1 h, 14 h, 24 h and 48 h) for 4 hours before fixation. Immuno-detection of phosphorylated form of histone H2AX was performed^20^. As shown in Fig. 1b, phosphorylated histone H2AX was detected in spheroids grown in PAM indicating that PAM induces DNA damage. Intense staining was detected in outermost region of spheroid treated by PAM in the case of time Ttrans = 14 hours. This staining was gradually decreases in the case of Ttrans = 24 h and was not detectable in spheroid grown in PAM for Ttrans = 48 h.

Together, these results indicate that PAM displays a genotoxic activity and inhibits growth. Furthermore, genotoxic activity appears to decrease along time and is barely detectable after 48 hours storage of PAM at 37 °C.

### DNA damage is dependent on the exposure time of culture media to plasma jet

**Figure 2:**
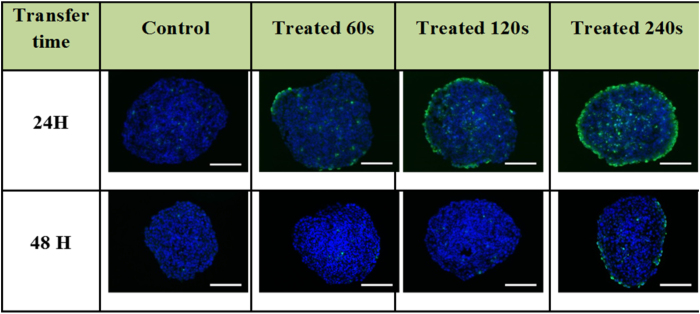
Genotoxic effect of PAM prepared following increasing exposure duration (Texp = 60 s, 120 s and 240 s). MCTS were transferred in plasma-activated medium after 24 h or 48 h, and fixed 4 h later. Immunodetection of the phosphorylated from of histone H2AX (phospho-H2AX, green) on 5 μm cryosections of control or treated spheroids. DAPI (blue) correspond to the Nuclei. Data shown are representative images from four independent experiments. Scale bar, 100 μm.

Exposure time (Texp) of the culture media to the He plasma jet is likely to be a key parameter in PAM activity on growth inhibition and DNA damage. To investigate this issue, culture media was exposed from 60 s to 240 s to He plasma jet. Spheroids were then grown in PAM and the DNA damage inducing effect was evaluated. Figure 2 displays representative cryosections of spheroids grown for 4 h in 3 types of PAM (Texp = 60 s, 120 s and 240 s) prepared 24 h and 48h before the exposure of the spheroids. In the case of time Ttrans = 24 h, DNA damage detected in the outmost region of HCT116 spheroids increase in a plasma jet exposure time (Texp) dependent manner. For Texp = 60 s, only few sparse fluorescent spots were observed indicating limited DNA damage while in contrast the staining is strongly intense in spheroid treated by PAM activated during 240 s. In the case of a transfer after 48 h DNA damage is observed only in the case of 240 s exposure time. Thus, these data indicate that plasma jet exposure produces chemically reactive species in the culture medium in a time exposure-dependent manner.

### H_2_O_2_ plays a major role in DNA damage of multi cellular tumor spheroids

Low temperature plasma jet induces complex kinetic reactions between gaseous plasma species and the culture medium leading to the generation of many aqueous chemically reactive species with long lifetime. We have chosen to emphasize the role of more particularly H_2_O_2_, which is one of these chemical species already highlighted in the literature^33^.

**Figure 3:**
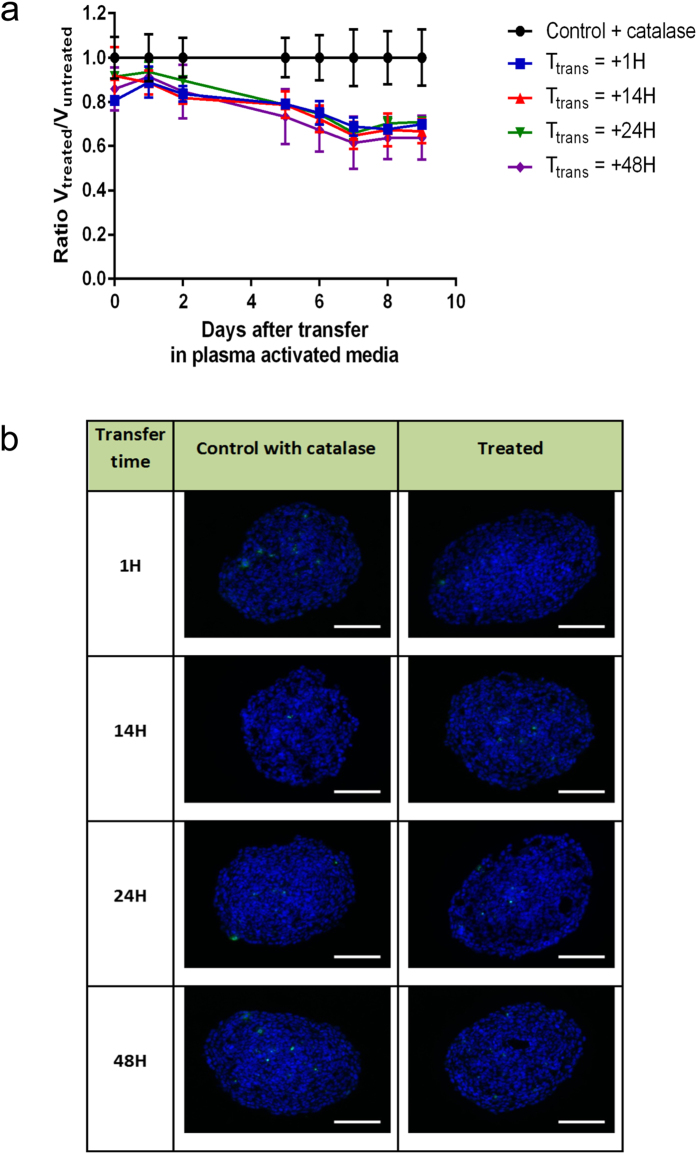
Growth inhibition and genotoxic effects of PAM in the presence of catalase. (**a**) Variation of the relative volume of HCT116 spheroids grown in plasma-activated media in the presence of catalase (30 μg.mL^−1^). Spheroids were cultured during five days in DMEM and subjected to plasma-conditioned medium at day 0 for several transfer time Ttrans. Data are shown as means ± SD from four independent experiments. (**b**) Genotoxic effect of PAM detected 4 h after transfer time of MCTS in PAM. Immuno-detection of the phosphorylation of the histone variant H2AX (phospho-H2AX, green) on 5 μm cryosections of control or treated spheroids. DAPI (blue) correspond to the Nuclei. Data shown are representative images from four independent experiments. Scale bar, 100 μm.

To this aim we first examined whether the putative effect of oxidative hydrogen peroxide molecules in PAM culture media could be neutralized by catalase addition, an enzyme known to catalyze decomposition of hydrogen peroxide into water and oxygen^34^. We first examined DNA damage of spheroid induced by PAM in the presence of catalase (30 μg.mL^−1^). Catalase was also added in culture media of control spheroid to observe a possible direct effect of catalase on spheroids. As shown in Fig. 3b, DNA damaging effect of PAM is fully reversed by the presence of catalase. In all transfer time conditions (1 h to 48 h), no growth staining of phosphorylated-histone H2AX was detected on spheroids. However, unexpectedly, a moderate growth inhibitory effect was still observed (Fig. 3a) even if the absence of DNA damage. Altogether, these data confirm that H_2_O_2_ is the major species involved in DNA damage observed in HCT 116 MCTS treated with PAM.

The generation pathway of H_2_O_2_ in PAM can be attributed to the reaction of short-lived ROS produced in liquid medium when impacted by the gaseous plasma species. The most important ROS that can be generated in the present case are hydroxyl radical, superoxide anion radical, and singlet molecular oxygen. In order to estimate the production rates of these species (OH^•^, 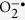 and O_2_(^1^Δg)) involved in H_2_O_2_ formation, different scavengers were added into culture media just before plasma exposure. Superoxide dismutase (SOD) is an enzyme that allows quenching of superoxide 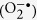 to following a redox cycle by using a metalo-organic complex of copper, manganese, iron or nickel^35^. The hydroxyl radical (OH^•^) can react with itself in aqueous phase to produce hydrogen peroxide following a free-radical recombination. To quench this reaction, D-mannitol^36^ is added before plasma treatment in culture medium. For a similar objective, L-histidine is added to the medium before treatment to quench singlet molecular oxygen^37^ (O_2_(^1^Δg)). The concentrations of the added scavengers in media before plasma jet treatment are summarized in Table 1.

**Figure 4:**
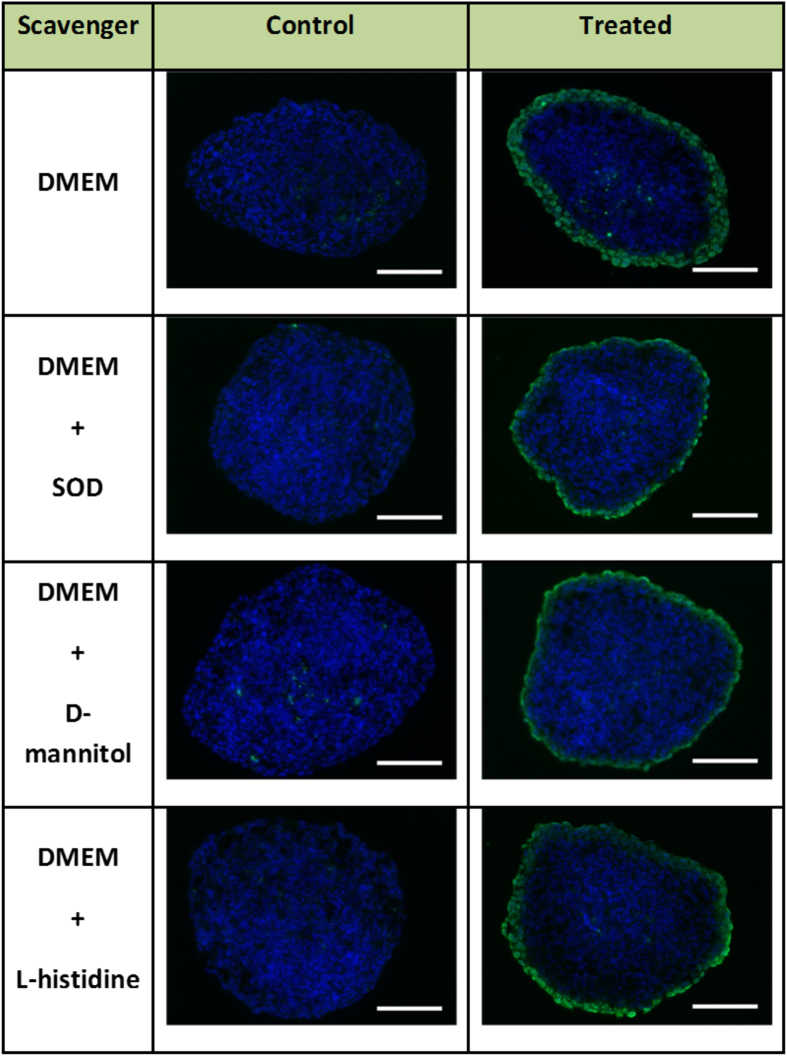
Genotoxic effect of PAM prepared of with free-radical scavengers. Immunodetection of DNA damage using antibodies against the phosphorylated form of the histone variant H2AX (phospho-H2AX, green) on 5 μm cryosections from control or treated spheroids fixed 4 h after transfer of MCTS in plasma-activated medium (Texp = 120 s and Ttrans 1 hour). DAPI (blue) correspond to the Nuclei. Data shown are representative images from four independent experiments. Scale bar, 100 μm.

In these experimental conditions we examined DNA damage after exposure to PAM. As presented in Fig. 4, DNA damage was still detected in PAM exposed spheroids, however the effect of PAM appeared limited to the outmost layer of cells in presence of SOD, D-mannitol , while the effect of L-Histidine was not obvious.

A detailed analysis of these images with the Image J software allows quantifying the percentage of spheroid volume in which DNA damage is detected upon exposure to PAM. The DNA damage volume was 33.6 ± 3.5% for spheroid grown in PAM, 22.8 ± 1.12% in PAM with SOD (scavenger of 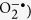, 23.5 ± 2.4% with D-mannitol (scavenger of OH^•^) and finally to 29.5 ± 1.6% with L-histidine (scavenger of O_2_(1Δg)). The DNA damaged spheroid volume is clearly dependent on scavenger type. Indeed, trapping of superoxide anion or hydroxyl radical reduces DNA damage of about 30% compared to the case of MCTS grown in PAM without scavenger. In the case of SOD this proportion is more important because SOD-catalyzed dismutase superoxide to product either molecular oxygen (O_2_) or hydrogen peroxide (H_2_O_2_). These results indicate that 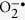 and OH^•^ are involved in the production of hydrogen peroxide in PAM. In contrast, given the limited reduction of DNA damage observed with L-Histidine scavenger, the singlet oxygen does not seem to play a significant role in the production hydrogen peroxide.

### Evaluation of H_2_O_2_ concentration produced in PAM by He plasma jet

**Figure 5:**
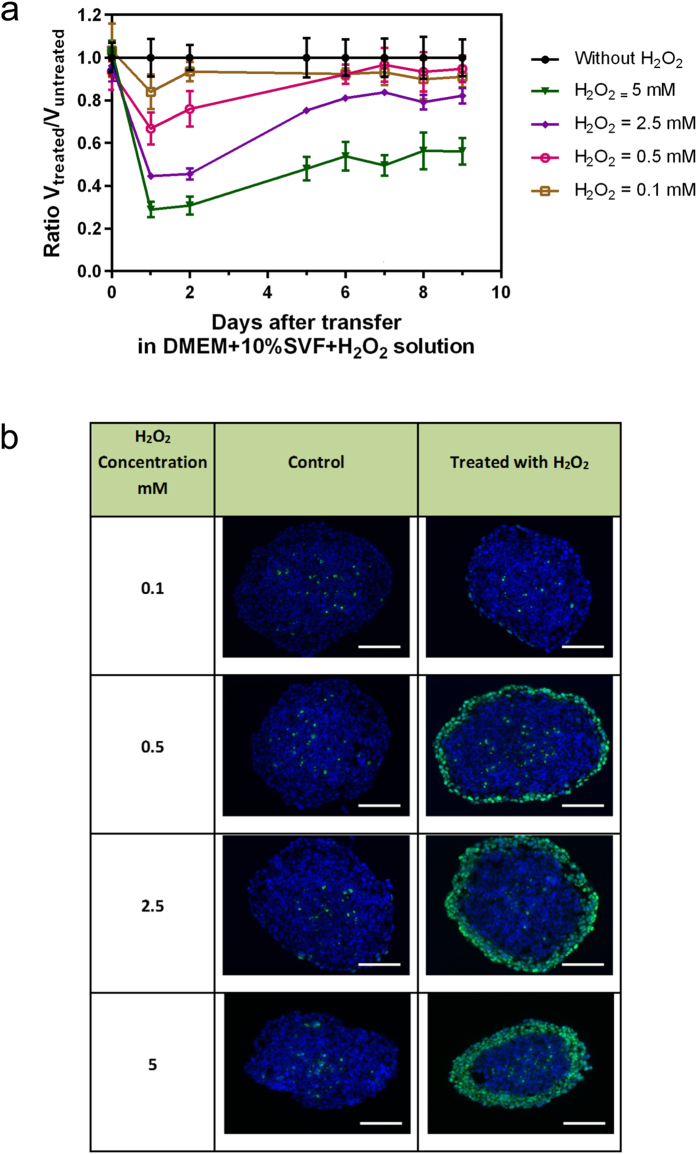
Growth inhibition and genotoxic effects of hydrogen peroxide exogenously added to culture media in MCTS. (**a**) The time-evolution of the growth of HCT116 spheroids after their transfer into culture medium in the presence of increasing concentration of exogenous hydrogen peroxide. Spheroids were cultured five days in DMEM and then transferred in culture medium containing H_2_O_2_ at day 0. Data are shown as means ± SD from four independent experiments (**b**) DNA damage due to hydrogen peroxide concentration added in culture media without any plasma treatment. Genotoxicity is analyzed by immunodetection of the phosphorylation of the histone variant H2AX (phospho- H2AX, green) on 5 μm cryosections of control HCT116 MCTS or MCTS for transfer time Ttrans 1 h. DAPI (blue) correspond to the Nuclei. Data shown are representative images from four independent experiments. Scale bar, 100 μm.

In order to confirm the involvement of H_2_O_2_ in the induction of DNA damage on MCTS cultured in the presence of PAM, we evaluated the effect of H_2_O_2_ addition (initial solution from 30 wt. % in H_2_O, Sigma) to culture medium without any prior plasma treatment. Both DNA damage and growth inhibition on the MCTS were examined. In the case of medium containing exogenous H_2_O_2_, two evolution phases of volume ratio were observed in Fig. 5a. The first one confirms a growth inhibition observed during the first day that is associated with dead cells detachment subsequent to DNA injury induced by H_2_O_2_. The second phase occurring systematically the second day and during which the spheroids have a greater growth dynamics than the control one (positive slope of daily volume ratio evolution of spheroids treated with H_2_O_2_). Figure 5b shows that exogenous H_2_O_2_ induces DNA damage in H_2_O_2_ concentration-dependent manner. Comparison of the extend of DNA damage observed in spheroid grown in PAM (Fig. 1b) or in the presence of added exogenous H_2_O_2_ (Fig. 5b) suggests that H_2_O_2_ concentration in plasma-activated culture medium for Texp = 120 s is between 0.5 and 2.5 mM.

This result demonstrates that the addition of exogenous H_2_O_2_ induces DNA damage the first day but this is not sufficient to obtain both DNA damage and growth inhibition of MCTS. This underlines that treatment of cancer cells with only exogenous H_2_O_2_ cannot stop growth inhibition thus underlying the central role of the other plasma byproducts generated in the liquid media.

### Growth inhibition and genotoxic activity of PAM are retained during 7 days of storage at either +4 °C or −80 °C

**Figure 6:**
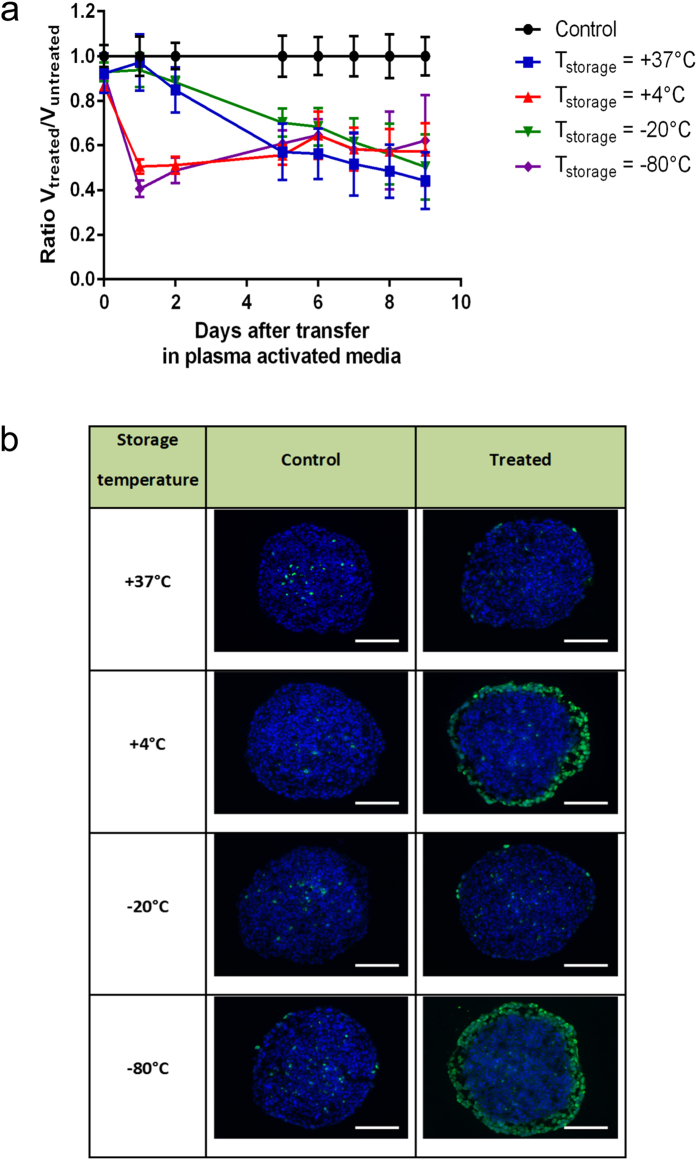
Growth inhibition and genotoxic effects of PAM stored at different temperatures. (**a**) Evolution of volume ratio of MCTS grown in PAM activated during Texp = 120 s and stored at different temperature during 7 days: +37 °C, +4 °C, −20 °C and −80 °C. Spheroids were cultured five days in DMEM and then transferred in PAM at day 0. Data are shown as means ± SD from four independent experiments. (**b**) Genotoxic effect of PAM detected by immunodetection of the phosphorylation of the histone variant H2AX (phospho-H2AX, green) on 5 μm cryosections from control spheroids or treated spheroids 4 h after transfer of MCTS in PAM (exposure time to plasma jet Texp = 120 s and time transfer inside PAM Ttrans = 7 days). DAPI (blue) correspond to the Nuclei. Data shown are representative images from four independent experiments. Scale bar, 100 μm.

Preservation of PAM growth inhibition and genotoxic activities as a function of temperature of storage was examined. To this aim, PAM was prepared after exposure to He plasma jet during 120 s, then stored during 7 days at different temperature conditions +37 °C, +4 °C, −20 °C and −80 °C, and used to grow spheroids for 4 hours. Figure 6b displays DNA damage detection 24 h after MCTS treatment with PAM stored at the indicated temperatures. Quantitative analysis of fluorescence images shows that about 36% of spheroid volume presents DNA damage with PAM stored at +4 °C. This volume is about 44% when PAM was stored at −80 °C. The ability of PAM to induce DNA damage was not changed after 7 days of storage at temperature of either +4 °C or −80 °C. In contrast, this genotoxic activity disappears in the case of PAM stored of +37 °C or −20 °C.

These results suggest that H_2_O_2_ concentration in PAM remains stable during at least 7 days of storage at +4 °C and −80 °C while H_2_O_2_ is certainly decomposed when PAM was stored at +37 °C and −20 °C. Growth inhibition effect displayed in Fig. 6a confirm DNA damage results. In the cases of PAM stored at +4 °C and −80 °C, the volume loss can reach about 50% due to cells detachment presenting DNA damages during the first day. The volume of spheroids treated with PAM stored at +37 °C and −20 °C can also reaches 50% but only after several days in contact with PAM (Fig. 6b). This inhibitory effect of PAM on spheroid growth remains similar whatever the storage temperatures suggesting that the concentration of the species responsible of growth inhibition does not depend on the PAM temperature storage.

## Discussion

In the present work, we analyzed the effects of plasma-activated media on the growth inhibition and DNA damage on HCT 116 colon adenocarcinoma multicellular tumor spheroids. PAM is generated by low temperature plasma jet using helium carrier gas operating in open air during several chosen exposure times (Texp varying between 60 s to 240 s). The spheroids are grown in PAM after different transfer times (Ttrans) chosen between 4 hours to 7 days.

We first confirmed and extended our previous findings^20^ concerning the growth inhibition of PAM on MCTS. We reported a strong link between DNA damage of MCTS volume and the transfer time of PAM when it is firstly exposed to plasma during 120 s and stored at +37 °C. This genotoxic activity can be observed 24 h after PAM activation. In contrast, growth inhibition effect is also observed for all transfer times. These results indicate that the observed effects of PAM on multicellular spheroids is due to the accumulation of long lifetime chemically reactive species generated in PAM during plasma short exposure time (equal to 120 s). PAM efficiency on spheroids are therefore dependent on transfer time and exposure time. Similar dependence has been also recently shown by Mohades^38^ in the case of monolayer cultured (or 2D) model of carcinoma cell.

The quantitative analysis of fluorescence images has shown that the decrease of volume ratio can be correlated to the loss of peripheral spheroid cells displaying DNA damage as already underlined in our previous work^20^. The reduction in the volume ratio means that all the cells subjected to DNA damage will then detach from the spheroids to die either by necrotic or apoptotic pathway. Moreover, when there are minor and/or manageable DNA damage the microscopy observation do not show any cell detachment but growth inhibition is still observed. This confirms that cytotoxic species in PAM can induce cell growth inhibition without DNA damage.

In our previous work^20^, we demonstrated that reactive oxygen species (ROS) are involved in the DNA damaging effect detected in MCTS in the case of treatment by direct exposure to the plasma jet. The experiments reported here with and without catalase scavenger suggest that H_2_O_2_ is the main agent involved in the observed DNA damage. Furthermore, by comparison between the effect of non-activated medium supplemented with exogenous H_2_O_2_, the concentration of H_2_O_2_ produced in plasma-activated culture medium for Texp = 120 s was estimated to be between 0.5 and 2.5 mM. Fluorimetric Hydrogen Peroxide Assay Kit (Sigma) was used to validate the estimated concentration of hydrogen peroxide produced by plasma jet in culture medium. For 120 s exposure time, the measured concentration of H_2_O_2_ is 1.05 ± 0.09 mM. This result shows a good coherence between the two methods of hydrogen peroxide quantification.

In addition, in the absence of H_2_O_2_, moderate growth inhibition effect was still observed. These growth inhibitory effects can be likely associated with other radical species with a long lifetime formed during the generation of PAM when exposed to the plasma jet. Radical species may be involved in this growth inhibitory effect can include reactive nitrogen species (RNS)^39^ and/or the chemical change of amino acids present in the medium induced by plasma radical species^40^.

One important factor of the potential clinical application of PAM is its stability during storage. We reported here that PAM maintains its genotoxic activity during storage at either +4 °C or −80 °C during at least 7 days. The conservation of PAM genotoxic activity during this period of 7 days is in agreement with literature results^28^ in the case of the temperature storage of −80 °C but not for +4 °C which is the recommended temperature to store the manufactured hydrogen peroxide to avoid its decomposition. This can be due to the ability of H_2_O_2_ molecule to remain stable at +4 °C or at a temperature lower than −60 °C leading to the H_2_O_2_ crystallization state (according to the classical diagram phase^41^) thus avoiding its chemical decomposition and therefore preserving its capability to induce DNA damage to spheroids. This shows one more time the important genotoxic role played by H_2_O_2_ plasma induced in PAM. Furthermore, PAM stored during the same duration of 7 days has maintained also its growth inhibition properties. In conclusion, we demonstrated that PAM has both genotoxic and cytotoxic effects on HCT116 colon adenocarcinoma multicellular tumor spheroids when stored at appropriate temperature (e.g. +4 °C). Forthcoming work will have to be devoted to further investigate the identity of the genotoxic and cytotoxic aqueous species using electron spin resonance.

## Materials and Methods

### Low temperature plasma jet at atmospheric pressure

Low temperature plasma jet is generated by using dielectric barrier discharge previously described elsewhere^20^,^42^. In short, plasma discharges are produced in a quartz tube (4 mm and 6 mm for respectively inner and outer diameters) on which two aluminum tape electrodes having 20 mm width are wrapped and separated by 10 mm gap. Helium gas carrier is injected with a flow rate of 3 sl/min. The electric discharge is ignited by mono-polar square pulses 9 kV of voltage magnitude, 10 kHz of frequency and 1 μs duration of square pulse.

**Figure 7:**
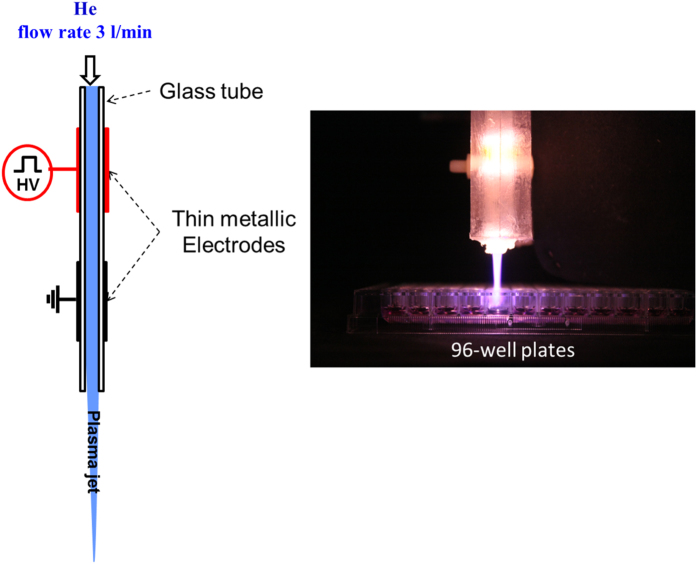
Experimental setup. In the left side: Illustrative set-up low temperature plasma jet using dielectric barrier discharge by mono-polar pulsed high-voltage power (voltage = 9 kV, repetition rate = 10 kHz, pulse width = 1 μs), and using helium flowing gas at 3 liter min^−1^ in the upstream side. In the right side: Preparation of PAM by exposition of 100 μL culture medium in 96-well plates to the low-temperature Helium plasma jet. Distance between culture medium and the top of the quartz tube is fixed at 2 cm.

Figure 7 displays a illustrative view of the plasma jet device and an example of plasma exposure of 100 μL culture medium in a well of 96-round bottom well cell culture plates. The gas temperature estimated around 30 °C using emission spectroscopy based on OH(A-X) molecular spectra. Furthermore, generation of ROS, such as hydroxyl radicals, singlet oxygen radicals, nitrogen oxide, and nitrogen, were confirmed by optical emission spectroscopy^20^.

### Cell culture and spheroids generation

HCT116 colorectal cancer cells (ATCC) were cultured in growth medium (DMEM (Invitrogen) supplemented with 10% Fetal Calf Serum (FCS) and 2 mML^−1^ glutamine and penicillin/streptomycin) in 5% CO_2_ humidified atmosphere at 37 °C^20^. Centrifugation method was used to generate spheroids in low attachment multi-well plates, as previously described^20^,^43^. In short, 500 cells/well were collected and distributed in poly-HEMA-coated 96-round bottom well plates. Following centrifugation (600 g during 6 min) the plates were placed in a humidified atmosphere of 5% CO_2_ at 37 °C. By using this technique single spheroids were obtained in each well and the size variation between spheroids is less than 10%. When the diameter of spheroids is about 400 μm (measured by using calibrated eyepiece reticule), the plasma treatment can be started.

### Immunofluorescence on spheroid cryosections

After PAM treatment, neutral-buffered formalin (Sigma) was used to fix spheroids during 2 h, the spheroids were rinsed with PBS (phosphate buffer saline) and stored at 4 °C^20^. After fixation, PBS with 15% and then 30% of sucrose was used to incubate spheroids at 4 °C during 24 h. The latter were embedded in Tissue-Tek (Sakura Finetek) and then cut in 5 μm-think cryosections. After a blocking step in PSB, 10% SVF, 0.5% Triton X-100, spheroid sections were incubated with antibodies against phosphorylated Histone H2AX (mouse monoclonal, Millipore, 1/500 one hour at 37 °C). After washes in PBS/0.1% Triton X-100 v/v, the secondary antibody was added for 1 h (anti-mouse conjugated with Alexa 488, Molecular Probes, 1/800, at room temperature). DNA was stained using 4′, 6-diamidino-2-phénylindole (DAPI)^20^.

### Image processing and analysis

As previously depicted^20^, epifluorescence microscope DM5000 (Leica) equipped with CCD camera (Roper COOLsnap ES) was used to collect fluorescence images of spheroid sections of 5 μm thickness. Software packages (Metavue and ImageJ) were then used to process fluorescence.

### Data statistics

The reported data correspond to the mean ± SD (standard deviation) of at least three independent experiments for the untreated spheroids and four independent experiments for the treated spheroids. Student’s test was used to perform statistical analysis.

### Production of plasma activated medium (PAM) and cell treatment

**Figure 8:**
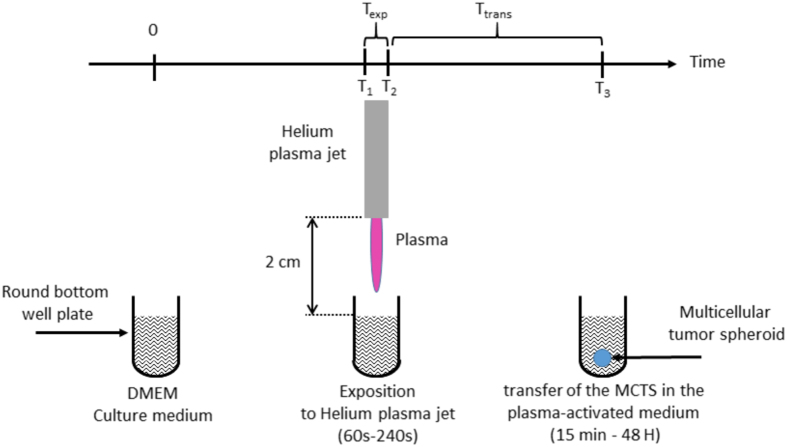
Main steps of the experimental procedure displaying (1) at time = 0, the well containing 100 μl of the liquid medium before the plasma exposure, (2) the preparation of PAM corresponding to the duration of the exposure of the culture media to low temperature plasma jet (Texp) and (3) the delay (Ttrans) between the end of plasma exposure and transfer of MCTS in this conditioned media.

Conditioned culture medium, designated by PAM, was produced through the exposure of cell culture media to low temperature plasma jet using helium carrier gas. Figure 8 shows a diagram depicting the experimental setup. The complete culture medium made from DMEM with 10% FCS and 2 mM.L^−1^ glutamine and penicillin/streptomycin. DMEM was exposed to the plasma jet in 96-wells plates (100 μL per well) for duration ranging from 60 s to 240 s (Time exposure, Texp). For data reproducibility, all plasma exposures were performed under the same experimental conditions (applied voltage, frequency, pulse duration and gas flow), same distance d (d = 2 cm) between plasma jet tube output and the upper-surface of liquid medium (see Fig. 8). A concealing plate (not illustrated in fig. 7) was always used to mask neighboring wells to avoid cross-exposure during the plasma exposure. 96-wells plates containing PAM were then stored in a humidified atmosphere of 5% CO_2_ at 37 °C.

HCT116 spheroids were transferred in PAM immediately after its preparation or after a selected delay subsequently referred as Time transfer Ttrans (Ttrans = 1 h, 14 h, 24 h and 48 h) (Fig. 8). The spheroid size is about 400 μm diameter when it is transferred in PAM. At that stage a proliferation gradient starts to organize with quiescent cells in the inner layers. No necrotic core is detected and will appear at later stages of growth.

## Additional Information

**How to cite this article**: Judée, F. *et al.* Short and long time effects of low temperature Plasma Activated Media on 3D multicellular tumor spheroids. *Sci. Rep.* **6**, 21421; doi: 10.1038/srep21421 (2016).

## Figures and Tables

**Table 1:** Concentration of free-radical scavengers added in culture medium before plasma treatment.

Scavenger	Trapped species	Concentration of scavenger
SOD	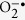	150 U/well
D-mannitol	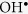	2.38 mM
L-histidine	O_2_ singlet	2.38 mM
